# GIMS—Software for asset market experiments

**DOI:** 10.1016/j.jbef.2015.02.001

**Published:** 2015-03

**Authors:** Stefan Palan

**Affiliations:** Department of Banking & Finance, University of Innsbruck, Austria; Institute of Banking & Finance, Karl-Franzens-University Graz, Austria

**Keywords:** Asset market, Experiment, Software, Double auction, Call auction

## Abstract

In this article we lay out requirements for an experimental market software for financial and economic research. We then discuss existing solutions. Finally, we introduce GIMS, an open source market software which is characterized by extensibility and ease of use, while offering nearly all of the required functionality.

Since the beginning of human history, trade has been an essential part of the lives of the human race and has played an important role in the development of civilization. Very early, centralized places of exchange were established to facilitate trade, promote competition, and thereby reap efficiency gains. Tremel (1969) for example writes about trade as early as in the neolithic (∼10000–3000 BC), and mentions fortified marketplaces in the bronze age (∼3000–1000 BC). The earliest comprehensive written records date from 11th century Europe (Casson and Lee, 2011).

Today, our globalized society is unimaginable without international trade. This is particularly evident in capital markets, where the greatest proportion of overall trading volume is handled by centralized marketplaces, with international traders. The value of the world’s stock capitalization alone amounts to $54.57tn.^1^ Recent years have underlined the relevance of well-functioning markets. Crises like the crash in 2000, following the bubble in prices of new economy stocks, and the real estate and banking crisis of 2007 through 2012, were accompanied by severe impacts on global economic activity and prosperity. These and other crises thus showcased the interconnected nature of asset markets and the real economy.

Despite the great relevance and enormous volume of trade handled by centralized exchanges, however, the economics discipline still lacks a thorough understanding of all the factors and processes driving market activity. The Sveriges Riksbank Prize in Economic Sciences in Memory of Alfred Nobel for 2013 constitutes a fresh and apt metaphor for this observation: While all three laureates contributed to the understanding of how asset prices form, at least two hold diametrically opposed views of how well real-world financial markets fulfill their function of revealing “correct” prices. Eugene Fama is a proponent of the Efficient Market Theory, positing that markets instantaneously, fully and correctly aggregate new information, such that market prices can at all times be seen as the best estimate of the assets’ true underlying values. Robert Shiller on the other hand argues that market prices are intrinsically biased and driven by human behavior, which causes systematic deviations from the benchmark of informational efficiency.

Such diametrically opposed points of view can only persist in a world where joint tests of market efficiency and a given asset pricing model are difficult, if not impossible (see for example the critique in Roll, 1977). This difficulty in judging the degree of market efficiency from empirical observations called for new scientific tools. Starting in the second half of the last century, experimental economics has begun to fill this gap. Using experiments where the researcher controls the market design, including the fundamental value of the asset being traded, the discipline has made substantial progress in identifying factors which inhibit and promote the correct functioning of markets. In particular, a multi-period asset market design introduced in Smith et al. (1988) has sparked a long line of publications extending our knowledge about the factors driving informational market efficiency. See Noussair and Tucker (2013) for an overview of the most important results from experimental asset market research in general, and Palan (2013) for a survey of the literature based on the Smith et al. (1988) paradigm. Overall, the literature of experimental asset market research has continued to grow for many years—a development which furthermore appears to be accelerating over time.

Nearly all of the (recent) papers studying experimental asset markets share their reliance on computer software to provide the market infrastructure. It is the aim of this paper to lay out the requirements for a high-quality experimental market software to cover a broad range of research questions, and to then propose a new software solution to address these requirements.

The paper is structured as follows: We start by laying out the requirements for a general-use software solution for asset market experiments in Section  1. Section  2 then gives a concise overview of existing software packages intended to serve this purpose. Section  3 presents a new package which, we will argue, addresses most of the requirements outlined in Section  1. Section  4 concludes, and the Appendix contains auxiliary information.

## 1 Requirements for experimental asset market software

We begin the body of this paper by laying out requirements for a general-use software solution for conducting asset market experiments. As such, we refrain from diving deep into the discussion of very specific solutions for one particular research question and instead outline a software which would be able to cover the bulk of the research done in this area. Nonetheless, one of the most important features of any research tool must be its comprehensive customizability, which alone renders it suitable to address not only current but also future research questions.

### 1.1 General requirements

#### 1.1.1 Complete, time-stamped data record

It is important for any scientific software to ensure that no data is ever overwritten or lost. Most experimental software packages save data in real time or at least multiple times during an experiment, thus reducing the risk of data loss due to e.g. power outages or hardware problems. Nonetheless, in many implementations of experimental markets, data is overwritten or not recorded. Frequently, precise times of events in the experiment are not recorded.^2^ As an example, take the case of the partial execution of a multi-unit limit offer in a market. It is important to ensure that both the original offer (including the original offer volume) and all transactions resulting therefrom (with their respective volumes) are saved and connected such that the mapping from original offer to subsequent transaction is clear.

#### 1.1.2 Customizability and extensibility

Software for economic experiments is subject to an important tradeoff: that between the ease of using and customizing it versus the degree of customizability and extensibility itself. On the one end are solutions like Charlie Holt’s VeconLab (veconlab.econ.virginia.edu), where parameters can be set with a few clicks, while at the same time offering only minimal customizability.^3^ On the other end are solutions custom-programmed for a specific research question. In between, there are a handful of solutions which feature some non-modifiable background components (e.g. networking, data management, etc.), while leaving the most important aspects of the programming open to modification.

#### 1.1.3 Reliability and lifetime

Since branches of scientific research frequently remain of interest for many years, it is desirable for a software solution to have a similar potential life. This criterion is more likely to be fulfilled by software platforms which are in widespread use and continuous development outside of the institution developing the specific research application.

A second important criterion is the reliability of the software in daily use. Solutions which are prone to hang or crash are of little use in a research laboratory. Furthermore, it is highly desirable for the software to have the ability to recover from the loss of connection to, or crashes of, client computers. In any case, the software should continuously write all collected data to the disk, such that in the event of a malfunction at least partial data from the current session (up until the crash event) can be recovered.

#### 1.1.4 Non-standard hardware and experiment designs

While the majority of economic market experiments are run in dedicated laboratories, with between 6 and 14 student subjects, taking no more than 2–3 h, some researchers have conducted studies deviating significantly from this outline. Teaching experiments nowadays frequently allow students to use their own hardware (i.e., to access the market via web browsers on laptops, tablets or smartphones). Other experiments run over the internet, without the need for traders to be physically present in the same room or building. Some experimenters have furthermore conducted markets running for several weeks at a time, or employing more than 300 traders (cp. Williams, 2008). An experimental software should therefore ideally be capable of running both in an experimental laboratory and over the internet, and on a wide variety of different devices. Furthermore, it should offer the possibility of comfortably running relatively short experiments (a few minutes) as well as considerably longer ones (multiple weeks).

#### 1.1.5 Cost

Any software for research should be as affordable as possible in order to enable even scarcely endowed research institutions to participate in the scientific dialog. This speaks for e.g. open source software. An even bigger factor than the cost of purchasing software, however, frequently is the cost of its customization for a specific research question. Here, software which is based on a widely employed platform, where programmers and support are broadly available, is to be recommended.

### 1.2 Requirements regarding the market mechanism

#### 1.2.1 User-friendly interface

The interface is one of the most important elements of an experimental software, yet at the same time it is difficult to make general statements about what constitutes good interface design. Generally speaking, it is beneficial for an interface to be as uncluttered and clear as possible in order to facilitate subjects’ understanding of the interface, as well as their grasp of the goings-on in the market. Relevant information should at any time be clearly visible, while additional, less relevant information may be concealed until requested by the subject. In the case of applications where subjects’ response time is critical, it is furthermore important that the interface be optimized for speed. Finally, the interface should scale well to different screen resolutions, since laboratories frequently differ in the hardware and settings used.

#### 1.2.2 Choice between single- and multi-unit trading

The market mechanisms employed in experiments reported in the literature fall into two categories—those which allow subjects to submit only single-unit and those which allow subjects to submit multi-unit orders to trade. Bloomfield et al. (2009) for example use single-unit trading, mainly in an effort to simplify order submission. Hornung et al. (forthcoming) allow multi-unit order submission with the intention of allowing subjects to trade more “realistic” amounts of cash and assets.

#### 1.2.3 Single or multi-period trading with or without wealth carryover

Researchers use single or multi-period experiments. In the latter, there may be a fixed ending period or one which is determined during the course of the experiment (e.g., by coin flip at the end of every period). Furthermore, in multi-period experiments, some designs require asset and cash balances to be reset from one period to the next (see e.g.,  Palfrey and Wang, 2012), while others require them to carry over from one period to the next (e.g.,  Lin and Rassenti, 2012).

#### 1.2.4 Possibility to trade in multiple markets or over the counter

While most asset market experiments are limited to trading in a single asset, some offer subjects the possibility to trade in more than one asset (e.g.,  Kleinlercher et al., 2014, Childs and Mestelman, 2006). Similarly, the growing importance of over-the-counter trading and dark pools reinforces the importance of offering multiple trading outlets. When such a functionality to trade in multiple markets is present, interface design becomes even more relevant than for a single market design. In some experiments, multiple markets are displayed side-by-side on the trading screen, while in others subjects can switch between different markets. In addition to trading in multiple markets of simple assets, some experiments (e.g.,  Jong et al., 2006, Palan, 2010) allow subjects to trade derivatives on the main market’s asset.

#### 1.2.5 Parameter specification

Asset market experiments differ in how parameters like the initial endowments, possible dividend amounts or signals about the fundamental value of the traded asset are specified. While some designs call for these parameters to be randomly drawn by the software, others require the option to let the experimenter pre-specify them. Furthermore, in the case of complex parameter structures (e.g. different, pre-specified signal sets for different treatments; or parameters derived from the outcomes of a previous experiment), it is helpful if the experimental software allows for importing individual parameter values or entire sets of parameter values.

#### 1.2.6 Designated trader roles

Some experiments assign designated trader roles to their subjects. Instead of subjects being allowed to both buy and sell within the same period, designated buyer (seller) subjects are allowed only to buy (sell). See Smith (1962) for one of the most famous examples of such a design.

#### 1.2.7 Order validation

The area where, in our experience, a great number of experimental asset market software solutions are lacking is in order validation. Some programs allow the submission of crossing orders, i.e. submission of a bid (ask) with a price higher than the price of the best outstanding ask (bid). Others allow subjects to accept other than the best bid and ask offers. Some do not even rule out churning, i.e. the practice of subjects trading with themselves. The latter drives up trading volume and carries the risk that subjects–by trading with themselves at off-equilibrium prices–try to manipulate others into submitting orders with biased prices. In contrast to these examples, experimental asset market software should optimally enforce the following rules:

• Subjects may not spend more than their cash plus any margin available to them.
• Subjects may not sell units of the asset exceeding their inventory plus any short-selling capacity available to them.
• The price of a new bid (ask) shall not exceed (be lower than) the price of the lowest (highest) outstanding ask (bid) price.
• Subjects may not accept bids (asks) with prices lower (higher) than that of the highest (lowest) priced bid (ask) currently outstanding.
• Subjects may not trade with themselves.

Furthermore, some markets implement a bid–ask-improvement rule, i.e. a rule that any new bid (ask) must have a price higher (lower) than the best outstanding bid (ask). Finally, it may be desirable to limit the number of bids and asks any individual subject may have outstanding.

#### 1.2.8 Order types and priority

The minimum requirement for any market software is to admit limit orders. Apart from this, it is common to permit the submission of market orders in continuous double auction markets, while less so in sealed bid–ask call auction markets. Furthermore, some research questions require more advanced order types, like stop loss or iceberg orders.

Markets also differ with regard to the order priority when it comes to price determination and settlement. In continuous double auction markets, price–time priority is usually sufficient to establish a clear order of trade execution and settlement. In call auction markets, the situation is more complex. While it is common for international exchanges to commence the price determination by finding the price which maximizes trading volume (i.e., number of assets traded), the subsequent steps in the case where multiple prices fulfill this first criterion differ between exchanges. Table 2 presents the price determination mechanisms at a sample of international exchanges which publish them.

For most asset market experiments, the precise determination of priority in price setting and order execution is not part of the assumptions underlying the research question(s). Nonetheless, we consider it important to clearly report the precise mechanisms used (which is frequently not done). Of great importance may also be the speed of price determination and order settlement in call auction markets. It is vital that this procedure be fast enough to ensure fluid operation of the experimental market.

#### 1.2.9 Algorithmic trading

Treleaven et al. (2013) report for 2011 that more than 73% of U.S. equity trading volume was due to algorithmic trading (trading by “robots”). While experimental research has so far largely ignored this topics, the increasing importance of algorithmic trading for global security markets is likely to change this situation in the future. For this reason, the possibility to implement robot traders is a very desirable feature in any asset market software.

## 2 A brief overview of existing solutions

Many researchers conducting asset market experiments program proprietary markets. Due to missing data about these types of programs, and their unavailability to the wider research community, it is neither possible nor desirable to discuss each of them. We therefore limit ourselves to discussing publicly available solutions which have already been used in multiple published experiments. Unfortunately, at the time of our writing, development of a number of software platforms which were or still are publicly available has ceased, such as in the case of Aton (www.aton.com.au/activeexperiments.html), JessX (jessx.ec-lille.fr), Marketscape (marketscape.caltech.edu), Regate (www.gate.cnrs.fr/perso/zeiliger/regate/regate.htm), Weblab (github.com/tomrutter/WebLab) and Zocalo (prediction markets, zocalo.sourceforge.net). For other platforms which are still under active development, we were unable to find any mention of an asset market application: BoXS (boxs.uni-bonn.de), Coral (code.google.com/p/coral-econ), jars (github.com/s-plum/jars), Multistage (multistage.ssel.caltech.edu:8000/multistage) SWiEE (swiee.econ.unito.it) and Willow (econwillow.sourceforge.net). Finally, a few asset markets out there are unsuitable for research due to their extremely limited customizability: EconPlay (www.econplay.fr), Moblab (www.moblab.com) and Veconlab (veconlab.econ.virginia.edu). We are left with five publicly marketed experimental asset market software solutions under active development. We present them in the following sections. Prior to that, Table 1 gives a first overview of the main features of the five existing software packages plus the new solution presented in this paper.

### 2.1 EconPort MarketLink^4^

MarketLink was developed at the University of Arizona and is currently being supported from Georgia State University. It is based on Java and experiments can be run over the internet. The software allows for conducting continuous double auction and sealed bid–ask call auction experiments with limit and (optionally) market orders. Subjects can be assigned a fixed role–either buyer or seller–or can be allowed to both buy and sell. Trading can either be restricted to a single unit of the asset per order or can allow for multiple units. The costs and values of the asset can be induced individually per trader, or can be set equal for all market participants. Additional options are:

• Up to three simultaneous markets, with individual traders acting in all or in a subset of the available markets.
• Possibility to add taxes, subsidies, price controls and externalities.
• Possibility to visualize results using tables and graphs.
• Possibility to implement a bid–ask improvement rule.
• Periods can be independent or cash and asset balances can be carried over between consecutive periods.
• Optionally, traders can be granted the possibility to invest into changing their future marginal cost structure.

### 2.2 Flex-e-markets^5^

Flex-E-Markets is web-based software originally developed at Caltech. It offers both continuous double auction and sealed bid–ask call auction functionalities. It is based on javascript/HSQL but allows also for the use of MySQL. Given this use of current web technology, it should be relatively easy to find capable programmers to administer and modify Flex-E-Markets, although the possibility for modification is limited as the software is not open source. However, Flex-E-Markets provides mainly a trading interface and allows for much of the transaction-handling to be accomplished outside of it, by external programs. Its features include:

• Possibility to specify a maximum number of units a trader may sell short.
• Possibility to levy a transaction tax on each trade.
• The software allows for running multiple markets in parallel, including private markets where orders have to be addressed to specific counterparties.
• Combination orders: Set of multiple individual orders which have to be executed simultaneously.
• Activity and withdrawal limits: Maximum numbers of times a trader can submit and cancel a bid in an auction, respectively.
• Wealth can be set to carry over between different markets.
• Traders can be allowed to use an electronic chat to communicate with each other.
• Possibility to display a price chart.
• Possibility to display the indicative price which would obtain in a call market if it were cleared immediately.
• Algorithmic traders (robots) can be programmed in the Python programming language.
• Export of results and configurations in xml and/or csv format.
• APIs for connecting with other applications which may both retrieve and update market data using HTTP requests.
• Running markets with multiple periods is possible but requires manual payoff calculation and deletion of outstanding orders.

### 2.3 jMarkets^6^

Developed by the Social Science Experimental Laboratory at Caltech, jMarkets is open-source software based on Java. It features support for multiple simultaneous markets using continuous double auction and sealed bid–ask call auction institutions. The order book can be either open or closed, and traders can be either buyers, sellers, or both. jMarkets supports customized payoff and bankruptcy handling and allows for short-sale constraints and bankruptcy cutoffs. Cash and assets can be carried over from one period to the next, or can be converted into profits for the traders and re-initialized in new periods. jMarkets is optimized for large markets and supports experimentation over the internet. Additional features include:

• Real-time market updates: Security and cash holdings are updated automatically on client interfaces and changes in the order book are highlighted.
• Client re-authentication: A client can re-join a running session at any time by logging in again, whereupon holdings and standing offers are automatically restored.
• Personal orderbook: The screen displays a table showing all outstanding orders submitted by the client for each security in the current period.
• Transaction table: The screen displays a table showing the current period’s transactions the client was involved in.
• Earnings table: The screen displays a table showing how much the client earned in each period on each security, including the total payoffs.
• Real-time price chart: A price chart on the client interface displays price movements of each security in real time.
• Best price buttons: Best buy/sell buttons for each security allow traders to quickly hop to the best offers on either side of the market in order to conserve time.
• Past order and transaction caching: Both order and transaction panels can optionally be set to retain past trading period data.

### 2.4 Rotman Interactive Trader^7^

Developed by the Rotman School of Management at the University of Toronto, the Rotman Interactive Trader offers a highly customizable interface for both sealed bid–ask call auction and continuous double auction trading. It is structured as a client/server application and intended for use over the internet. The software supports various order types and offers facilities for implementing transaction costs. It also allows traders to link data to Microsoft Excel™ in real time and offers a built-in risk management system. The software is set up to allow for the simulation of equities, fixed income securities, options, futures and commodities, and it allows for the implementation of computerized agents filling the roles of noise traders, liquidity traders, informed traders and option traders. Finally, the Rotman Interactive Trader can announce–to all or only a subset of traders–news based on a predetermined timetable, or input by the experimenter. The software platform and associated simulation cases are available under a campus license.

### 2.5 SoPHIE Labs double auction asset market^8^

Based on the SoPHIE platform (Hendriks, 2012), the asset market add-on developed by SoPHIE Labs allows for conducting double auction experiments with a single asset. It offers an open order book, a list of transactions and a price chart displaying offers and transactions. All of these display elements are optional. Traders can submit and cancel bids and asks for a single unit of the asset, limited only by their budget of experimental cash and units of the asset. The software supports multiple languages. It is pre-configured for English and German, but other languages can be added by modifying a single text file. Since the solution is based on common web infrastructure like PHP/MySQL, it is highly extensible, and there is a deep pool of programmers available nearly worldwide. Furthermore, it is well-suited for conducting internet experiments and offers a direct integration of Amazon Mechanical Turk.

## 3 The Graz–Innsbruck Market System

The Graz–Innsbruck Market System (GIMS) is open-source software, developed to offer an extensively customizable infrastructure for market experiments. Technically, it is a z-Tree program, running on the z-Tree platform of version 3.4.2 and later (Fischbacher, 2007) and requires a minimum screen resolution of 1024×768 pixels but is optimized for wide-screen displays. The following sections give an overview of its built-in features. The current version of the software, including detailed documentation, can be downloaded from academic.palan.biz/gims.

### 3.1 General features

GIMS offers pre-programmed facilities for continuous double auction and sealed bid–ask call auction market experiments. In addition, it allows for the elicitation of several types of subject characteristics. In particular, experimenters can run the cognitive reflection test (CRT) ofFrederick (2005) and elicit the Holt and Laury (2002) risk aversion measure before or after the market phases. Furthermore, the software can be set to require subjects to answer financial literacy questions from van Rooij et al. (2011) at the end of the experiment. Finally, the software package includes a questionnaire eliciting subjects’ addresses, socioeconomic data, risk-attitudes, problems encountered during the experiment, and any other comments subjects might want to make. Appendix A.1 provides detailed information on all options for the elicitation of subject characteristics.

The following features characterize the software package:

***Multi-language support***. The software is currently capable of supporting experiments in English and German. Extending it to other languages requires the translation and entry of the new texts into all elements of the software required for a given experiment.

***Complete record***. The timing of every event in the experiment (subjects’ movement through the experiment, subject input, etc.) is recorded with millisecond precision in a single, unified timelog table. This timelog functionality can be extended to any part of the software modified or added for a specific research purpose. Furthermore, no data is ever overwritten. However, due to limitations of the z-Tree platform, data is written to disk only at the end of a period. While rare, this does admit the risk of losing the data of the current period due to e.g. the loss of electrical power or a hardware problem on the server computer.^9^ Nonetheless, the platform offers the possibility of exporting the state of play into tab-delimited files on disk at any point during the experiment.

***Experimenter screen***. Throughout the entire experiment, the experimenter can view market proceedings and can at all times (i) move all subjects to the next stage of the experiment, (ii) move subjects to the next period, or (iii) terminate the experiment. This feature is especially valuable in that it significantly reduces the time required for testing. Furthermore, due to this inbuilt support for experimenter supervision, it is easy to program experimenter interventions where desired. For example, it would be straightforward to allow the experimenter to enter the result of a random draw conducted with an offline randomization device like, e.g., a die or bingo cage.

***Non-standard hardware and experiment designs***. In line with z-Tree’s capabilities, GIMS’ suitability for non-standard hardware and experimental designs is limited. It requires a Microsoft Windows™ desktop or server operating system (Windows NT 3.51, NT 4.0, 2000, XP, Vista, 7, 8) on both the server and the client computers and can thus not be run on e.g., smartphones. Furthermore, while z-Tree can be run over the internet, configuring it to do so is not as straightforward as logging in to a market through a simple browser interface. Finally, since z-Tree is intended as a platform for laboratory experimentation, experiments running over longer time periods (multiple days) are not feasible using GIMS.

***Cost***. GIMS is both free and open-source software. Furthermore, most economics laboratories are already set up to allow for experimentation in z-Tree, since this is the programming language most frequently used in economic experimentation. Note that the paper by Urs Fischbacher describing the software (Fischbacher, 2007) has already been cited more than 3700 times according to Google Scholar, and more than 1200 times according to IDEAS, making it the most frequently cited article in *Experimental Economics*. Many universities teach z-Tree as part of their curriculum, and there are summer schools and workshops for students (and faculty) of universities which do not. The community has also created a number of free tools facilitating the import and/or analysis of z-Tree output in statistics packages like Microsoft Excel™, Stata™, and R. Finally, GIMS allows for replicating–with minimal effort–many of the experiments reported in the literature. Appendix A.2 discusses for a number of published economic experiments how they could be implemented in GIMS.

***Lifetime***. GIMS is built on the z-Tree platform, which has been under continuous development since 1995 and continues to be developed at the University of Zurich. GIMS itself is the product of several years of market experimentation by the groups in Graz and Innsbruck and currently plans are to continue developing and supporting it indefinitely. Even if development at the universities of Graz and Innsbruck should at some point in the future be discontinued, the open source license^10^ allows practically unlimited development by any interested third party and ensures that all future versions of the software will in turn be provided under the same open source license. Furthermore, in the spirit of open source software development, the developers will include selected modifications and new features developed by third parties if so desired and if they are of broader interest to the GIMS user base.

### 3.2 Market mechanisms

This section starts out by describing the functionality and characteristics common to both the continuous double auction and the sealed bid–ask call auction institutions in GIMS, before discussing elements specific to each.

***Designated trader roles and multi-market trading***. GIMS currently does not offer the option of designated trader roles or of trading in multiple markets or over the counter. The reason is parsimony of the code in light of the relatively small number of recent experiments implementing these design features. Nonetheless, it would be easy to implement different roles by changing the role-dependent screen layout such that buyers (sellers) simply would not see the requisite buttons and fields to enter offers to buy (sell) or accept offers to sell (buy). Similarly, it is possible to implement multiple market trading by changing the screen layout and using market IDs for all orders and trades (the latter is already preconfigured), thereby separating activity in the different markets.^11^

***Automatic validation of orders***. GIMS offers all of the order validation features outlined in Section  1.2.7. They are implemented as follows: The software permits all individually valid orders to be entered into the order book. After each transaction, all outstanding orders are evaluated and, in case that they (again, each individually) can no longer be (fully) filled by the submitting subject, their volume is optionally reduced (multi-unit setting) or the order invalidated (single- and multi-unit setting). In other words, a subject can have orders outstanding which he or she could not all fill jointly, but every single outstanding order viewed in isolation is always feasible. The bid–ask improvement rule can be turned on or off by the experimenter, as can the feature to limit the number of concurrent outstanding bids and asks by any one subject.^12^

***Algorithmic trading***. GIMS currently does not offer the option of implementing robot traders. However, the z-Tree programming language does in principle support programming automated responses to market or timer-driven events. Nonetheless, researchers planning to focus on this topic may presumably find it more convenient to use Rotman Interactive Trader or Flex-E-Markets, which are pre-configured to accommodate such functionality.

***Other features***

• Choice between single or multi-unit trading. In the former, the volume of each order and trade is one unit, without need for (or even possibility to) enter a desired trading volume.
• Free choice of the number of periods. Even a random ending period is easy to implement given the pre-configured implementation of an experimenter screen, where the experimenter can enter, in every period, whether or not the experiment continues.
• Possibility to reset or carry over subjects’ assets and cash between periods.
• Possibility to pay subjects based on the cash balance at the end of every period or only for the cash balance at the end of the final period.
• Dividends, subject endowments and signals (e.g., signals about the asset’s fundamental value or the state of nature) can either be randomly drawn from a freely specifiable distribution, or can be imported from file.^13^
• The interface is designed such that the buttons and fields for order submission are grouped close together to accelerate order entry. When scaling beyond the minimum resolution, the software is designed to make optimal use of the additional screen space (e.g., scaling up the size of the chart, while holding constant the size of screen elements with fixed space requirements). The different screen elements are color-coded to help subjects’ orientation and facilitate the process of explaining the screen interface.
• The order book can optionally be set to display only the n best offers (according to price–time priority), where n is any positive integer. A trader’s own offers are always being displayed.

Finally, the software offers the choice between a continuous double auction and a sealed bid–ask call auction mechanism, or the use of both in succession in the same period. The following subsections provide more details on the features of these two institutions.

#### 3.2.1 Continuous double auction

**Fig. 1:**
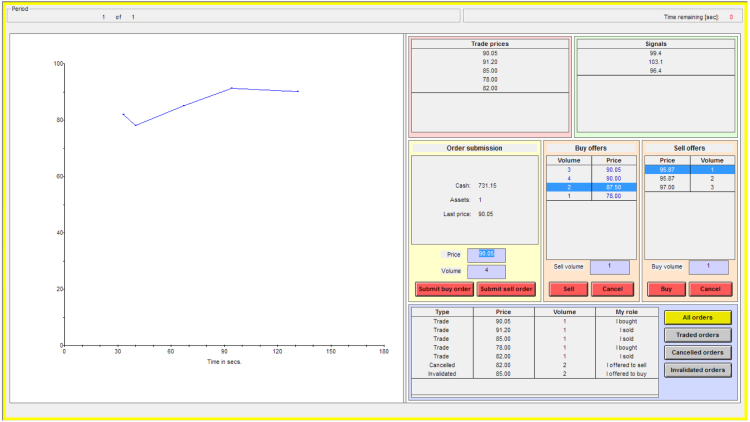
Continuous double auction market interface. The figure shows a screenshot of the continuous double auction market interface with multi-unit trading enabled. The display of the price chart and of the lists of transaction prices and signals is optional.

Fig. 1 displays a screenshot of the continuous double auction interface.

• Submission of limit and market orders. Execution occurs according to price–time priority. Stop loss, iceberg and other order types are currently not implemented.
• The best orders on both the bid and ask sides of the order book are always selected automatically to permit faster order acceptance.
• In a history window, every subject can display, for all non-open orders in the current period she either originated or accepted: (i) all of her orders, or only (ii) her trades, (iii) her canceled orders, or (iv) her orders which were invalidated by the computer because the subject’s cash or asset balance would have been insufficient to fill the order in case another trader had accept it.
• Transaction prices can optionally be displayed in the form of a table and/or a chart. The chart’s vertical axis rescales automatically to best accommodate the magnitude of the prices being displayed, while the horizontal axis rescales to accommodate the period length.
• The order book can be set to be emptied automatically after each trade.
• Subjects can be given the option to vote for ending the period early. If enabled, subjects can continue trading–even if they have voted–until the period ends due to all subjects having voted for an early end, or due to timeout.

#### 3.2.2 Call auction

**Fig. 2:**
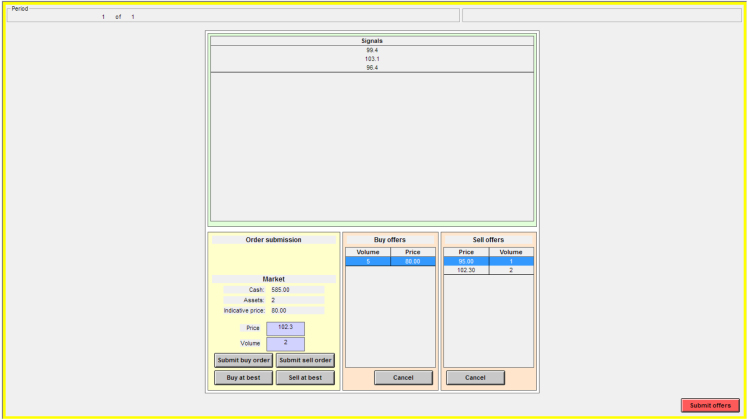
Sealed bid–ask call auction market interface. The figure shows a screenshot of the sealed bid–ask call auction market interface with multi-unit trading disabled. The display of the list of signals is optional.

Fig. 2 displays a screenshot of the sealed bid–ask call auction interface.

***Order types and priority***. GIMS allows for submitting limit and (optionally) market orders. The latter can be thought of as entering the price determination at very high (low) prices in the case of bids (asks). Note that allowing market orders introduces the risk of subject bankruptcy, since the finally resulting market price (at which market orders are executed) is unknown at the time of order submission and validation.

GIMS performs the following steps to determine the uniform auction price:

• Step 0: If the highest buy offer limit price is lower than the lowest sell offer limit price, no trade is possible. This extends to market orders. In this case, stop the price determination. Otherwise, continue.
• Step 1: If there is a single price maximizing the feasible trading volume, set the auction price equal to this price and stop the price determination. Otherwise, continue.
• Step 2: If there is a single volume maximizing price which minimizes the absolute oversupply, set the auction price equal to this price and stop the price determination. Otherwise, continue.
• Step 3: If there is a surplus of buy (sell) volume at all volume maximizing and absolute oversupply minimizing prices, set the auction price equal to the highest (lowest) of these prices and stop the price determination. Otherwise, continue.
• Step 4: Calculate the average of the highest and lowest volume maximizing and absolute oversupply minimizing prices and set the auction price equal to this average, rounded to the nearest tick. In the case of equal distance, round down to the nearest tick.

This mechanism replicates the price determination mechanism of NASDAQ. Steps 0–1 are the same for all markets listed in Table 2, and Step 2 is used by all markets except for the NYSE. At some point, most exchanges (except for NASDAQ) use a reference price (e.g., the previous closing price) in case earlier steps do not lead to a unique price. However, this is impractical in an experimental market, where there is no such price in the first period, and where–depending on the experiment–periods may be designed to be independent. It is for this reason that we have chosen to model the NASDAQ mechanism.

For trade settlement, GIMS features two price-order type-time priority rules—one in which limit orders whose price equals the auction price take precedence over market orders, and one in which the opposite is the case. The first is the procedure used at, for example, the National Stock Exchange of India’s “Emerge” market (NSE, 2014), while the second is employed by, for example, NASDAQ. (NASDAQ OMX Nordic, 2013). Trade settlement proceeds as follows:

• Step 1: Sort orders by priority.
  – If set to give market orders priority, then priority is: Better than equilibrium limit orders, market orders, at equilibrium limit orders.
  – If set to give limit orders priority, then priority is: Better than equilibrium limit orders, at equilibrium limit orders, market orders.
  – Within better than equilibrium limit orders, priority is by price; within all categories, final priority is by order of submission (earliest submission time equals highest priority).
• Step 2: Transact orders against each other in order of their priority until no transactable bid–ask pairings remain.

***Other features***

• Possibility to let subjects submit a single bid and ask pair, or entire bid and ask schedules, with or without limits on the number of individual bids and asks.
• Possibility to display the indicative price (i.e., the price which would obtain if the auction were closed immediately), updated at freely specifiable intervals.
• Relatively fast execution; price determination and order settlement for a 24-seat lab takes less than a second.^14^

## 4 Discussion and conclusion

The paper presents the requirements for a software for asset market experiments, suitable for a wide range of research applications. At the moment, most researchers or research teams develop their own, custom-built solutions. One problem with this approach is the inefficiency caused by the parallel development of similar applications. The cost of time and resources spent on the programming and testing is non-trivial and could instead be spent on running additional sessions or experiments. Furthermore, the programs custom-built for individual researchers or teams are frequently not made public (except for where the journal the results are published in requires submission and publication of software). Given this fact, it is not inconceivable that some instances of reported differential outcomes in apparently similar contexts may be owing to overlooked differences in the market software being used. It is for example common in experiments following the Smith et al. (1988) paradigm to report normalized trading volume. At the same time, the placement and thus the distance between buttons and entry fields on the screen differs between software solutions. Also, some packages allow subjects to accept an outstanding offer simply by clicking a “Buy” or “Sell” button, while others require them first to select the best outstanding bid or ask, or even to enter a desired volume. It can hardly be surprising, then, that trading volume and the pace of trading might be higher in experiments using the former design than in ones employing the latter. While this is only one example, we argue that standardization would reduce the number of auxiliary hypotheses experimental findings–and especially comparisons of findings from different experiments–are based on.^15^

We present GIMS as a possible candidate for such a standardized solution. In addition, we offer extensive documentation including examples of how to implement features and modifications exceeding the current capabilities of the software. By offering the software and the documentation as a free download, we wish to further the democratization of the study of asset markets. It is our intention to lower the cost of entry into this branch of research, and conserve scarce resources for researchers who choose to use it.

## Figures and Tables

**Table 1:** Market software feature overview. The table presents an overview of the main features of the experimental asset market software packages discussed in this paper.

Feature	EconPort MarketLink	Flex-E-Markets	GIMS	jMarkets	Rotman Interactive Trader	SoPHIE Labs
**Market**						
Double auction	X	X	X	X	X	X
Call auction	X	X	X	X	X	-
Indicative price display		X	X			-
Market orders	X		X		X	-
Multi-unit trading	X	X	X	X	X	-
Multi-period markets	X	a	X	X	X	
Multiple markets	X	X	b	X	X	-
Designated trader roles	X		b	X		
Price chart	X	X	X	X	X	X
Algorithmic trading		X	b		X	
Transaction tax	X	X	X		X	
Short selling		X	X	X		
**Other**						
Web-based	X	X	-	X	X	X
Import of configuration data		X	c			
Multi-language support			X			X
Open source		-	d	X		
Direct cost		-	-	-	USD 9000 p.a. (e)	EUR 1680 p.a. (f)

Empty cells indicate functionality which was not listed as being available in the documentation the author could obtain. Cells with a hyphen indicate functionality known not to be available. Cells with “X” indicate existing functionality. Cells with lower-case letters indicate more information in the notes below.

a Requires some manual input.

b Possible, but not built-in (requires some programming).

c Partly possible.

d The GIMS software itself is open source, while the z-Tree platform it runs on per se is not.

e Price for a one year campus license, as of 21.04.2014.

f Price for one year basic hosting plus 12 months of the asset market add-on, as of 12.08.2014.

**Table 2:** Call auction price determination mechanisms. The table lays out the rules used for determining the auction price in the call auctions of various global exchanges. The criteria are cumulative. For example, the second step at the ASX searches for the single volume-maximizing limit price which minimizes order imbalance.

Step	ASX^a^	BSE India^b^	NASDAQ^c^	NSE Emerge^d^	NYSE^e^	XETRA^f^
1	Single volume-maximizing limit price	Single volume-maximizing limit price	Single volume-maximizing limit price	Single volume-maximizing limit price	Single volume-maximizing limit price	Single volume-maximizing limit price
2	Minimum order imbalance	Minimum order imbalance	Minimum order imbalance	Minimum order imbalance	Price closest to the previous closing price	Minimum order imbalance
3	Highest (lowest) price if bid (ask) surplus for all such prices	Price closest to the previous closing price	Highest (lowest) price if bid (ask) surplus for all such prices	Reference price		Highest (lowest) price if bid (ask) surplus for all such prices
4	The two prices where buy and sell pressure change are compared to the reference price. If the latter is at or above (below) the higher (lower) of these two prices, then the highest (lowest) price is the auction price. If it lies between these two, the reference price is the auction price.		Average between the highest price with positive, and the lowest price with negative imbalance, rounded to the nearest tick and rounded down in case of equal distance.			Reference price

a The Australian Stock Exchange; see Comerton-Forde and Rydge (2006).

b The BSE; see BSE (2013).

c The National Association of Securities Dealers Automated Quotation; see NASDAQ OMX Nordic (2013).

d The National Stock Exchange of India’s Emerge market segment; see NSE (2014).

e The New York Stock Exchange; see NYSE Euronext (2011).

f The exchanges using the XETRA market system, e.g. the Frankfurt Stock Exchange and Vienna Stock Exchange; see Deutsche Börse AG (2013).

## Appendix

### A.1 Elicitation of subject characteristics

Experimenters can optionally elicit measures of subjects’ willingness to reflect before providing answers to mathematical/logical questions, of their risk preferences, and of their financial knowledge and skills. Far from developing new instruments for these tasks, GIMS offers tests which have previously been employed (and developed) for this purpose in experimental economic research.^16^

#### A.1.1 Cognitive reflection test

The CRT of Frederick (2005) is an unincentivized test consisting of three mathematical/logical questions, each of which has one obvious, but wrong, answer, and a correct answer which for most subjects requires some extra cognitive attention. It has been employed in many economic studies and high scores on this test (i.e., a higher number of correct answers) have been linked to, among other things, better performance in markets (Breaban and Noussair, 2014) In GIMS, the CRT can be administered before or after trading.

#### A.1.2 Risk aversion test

The lottery choice task of Holt and Laury (2002) presents subjects with ten pairs of lotteries (one each relatively safer, and one relatively more risky) with increasing expected values. It records the pair at which a subject switches from the relatively safer to the relatively riskier option as a measure of the subject’s risk aversion. The version implemented in GIMS enforces a single crossing point, thus ruling out inconsistent behavior. The lottery choice task can be administered before or after the market phases. Subjects’ payoff is determined by randomly selecting an option pair and then playing out the lottery selected by the subject for this pair. The possible payoffs are by default set to equal the nominal payoffs in the baseline of Holt and Laury (2002), using whatever unit of real currency is employed in the experiment. Naturally, these can be modified.

#### A.1.3 Financial literacy test

The financial literacy questionnaire of van Rooij et al. (2011) consists of 16 questions of varying difficulty. They test the basic understanding of interest compounding and inflation, as well as subjects’ knowledge of common financial instruments like savings accounts, bonds, stocks and mutual funds. In GIMS, subjects can be required to answer all 16 questions, or a freely specifiable subset thereof (Cheung and Palan, 2012 and Cheung et al., 2014 for example use a 10-question subset). The task can be left unincentivized, subjects can be paid a fixed amount for every correct answer, or they can be paid a fixed amount in case they have correctly answered a question randomly chosen after they have finished the test.

#### A.1.4 Exit questionnaire

The exit questionnaire is provided in German and English. While filling in the questionnaire, subjects cannot return to previous pages. Note that it is easy to customize the questionnaire by removing, modifying or extending it.

It starts with a form asking subjects to fill in their address and indicate their availability for further experiments (not reproduced here). The subsequent pages contain the questionnaire items below. For each entry, the variable name is listed in square brackets, while the captions and internal value coding are listed in parentheses. A horizontal line indicates a page break.

1. What is your gender? [Female]
  (1 = “Female”, 0 = “Male”)
2. What is your age in years? [Age]
  (Integer between 0 and 121)
3. Your nationality? [Nationality]
  (Open text)
4. What is your employment status? [Employment]
  (“Full-time”, “Part-time”, “None”)
5. If you are a student, what is your major? [Major]
  (Open text)
6. What is the level of the highest degree you are currently studying? [LevelDegree]
  (“Bachelor”, “Master”, “Doctor/PhD”, “Other”)
7. How do you see yourself: are you generally a person who is fully prepared to take risks or do you try to avoid taking risks? [RiskGeneral]^17^
  (0 = “Not at all willing to take risks” …10 = “Very willing to take risks”)
  People can behave differently in different situations.
  How would you rate your willingness to take risks in the following areas?
8. How is it …while driving? [RiskDriving]^17^
  (0 = “Risk averse” …10 = “Fully prepared to take risks”)
9. How is it …in financial matters? [RiskFinancial]^17^
  (0 = “Risk averse” …10 = “Fully prepared to take risks”)
10. How is it …in leisure and sport? [RiskLeisureSport]^17^
  (0 = “Risk averse” …10 = “Fully prepared to take risks”)
11. How is it …in your occupation? [RiskWork]^17^
  (0 = “Risk averse” …10 = “Fully prepared to take risks”)
12. How is it …with your health? [RiskHealth]^17^
  (0 = “Risk averse” …10 = “Fully prepared to take risks”)
13. How is it …your faith in other people? [RiskOthers]^17^
  (0 = “Risk averse” …10 = “Fully prepared to take risks”)
14. How often do you participate in extreme sports (Extreme sports include bungee-jumping, para-gliding, parachute jumping, gliding, rafting, diving and other dangerous sports)? [ExtremeSports]^18^
  (“Never”, “A few times”, “Occasionally”, “Often”, “Every chance I get”)
15. Do you currently smoke cigarettes? [Smoker]^18^
  (0 = “No” …1 = “Yes”)
16. If yes, how many cigarettes do you smoke in one day? [NumberCigarettes]^18^
  (Integer between 0 and 1000)
17. Did you ever make a mistake in entering a price, or clicked a wrong button? If so, please tell us exactly what went wrong and in what period: [Typo]
  (Open text)
18. Did you find the instructions in the market experiment clear and understandable? What if anything could be improved? [ClarityInstructionsMarket]
  (Open text)
19. Room for your comments: [Comments]
  (Open text)

### A.2 Usage examples

The following examples are intended to provide the reader with an impression of the amount of work which would be required to recreate various experiments from the literature using GIMS. In the interest of readability, the specific modifications of the code are omitted here. Section 5 of the GIMS manual (Palan, 2014) contains a number of design variations plus a step-by-step guide of the modifications to GIMS required to implement them.

#### A.2.1 Plott and Sunder (1982)

Plott and Sunder (1982) conduct oral double auction experiments with heterogeneously informed traders. These traders’ payoffs from the asset they trade are heterogeneous and state-dependent. To implement such an experiment in GIMS, a few modifications of the built-in features are necessary. First off, GIMS can optionally display signals to traders. To recreate Plott and Sunder (1982), the researcher would have to program the random draw of a state of nature and of signals derived therefrom. Furthermore, trader type-dependent payoff functions need to be specified. We estimate the time requirement for both at less than an hour.

#### A.2.2 Smith et al. (1988)

Smith et al. (1988) is one of the most famous papers in experimental asset market research, and the most prominent on long-lived asset markets, i.e., asset markets comprising more than one period without interim reset. The experiments listed therein can readily be run using GIMS, with only minimal modifications to the programming.^19^ The only feature requiring programming of a (single) new line of code is the implementation of the buyout value, whose size depends on the dividends drawn over the preceding periods (paying a fixed buyout value is possible in GIMS by default).

As for the market design, Smith et al. (1988) use a continuous double auction market with a bid–ask improvement rule and an order book displaying only the best bid and ask. Subjects are not allowed to buy on margin or to short sell the asset. Smith et al. (1988) let subjects vote for an early end to the trading period, which is realized if and only if unanimity is reached. All of these design features are also available in GIMS.

#### A.2.3 Smith et al. (2000)

Smith et al. (2000) vary the dividend payment modalities in their experiment. In their treatment A1, a lump-sum dividend is paid at the end of the last period in a market. In treatment A2, they pay a random dividend after each period, while in A3, they combine the two options by paying a random dividend after every period plus a lump-sum dividend at the end of the last. The implementation of the random buyback value requires programming a similar line of code as discussed in Appendix A.2.2, while the remaining functionality is implemented in GIMS by default.

Note that certain–as opposed to random–dividend payments, as implemented in Porter and Smith (1995), are also already built into GIMS. So are lottery dividends as in Ackert et al. (2009) and Ackert et al. (2012).

#### A.2.4 Haruvy and Noussair (2006)

The main treatment variation in Haruvy and Noussair (2006) is to endow subjects with the ability to sell the asset short. In treatments QL3 (QL6), subject’s short selling capacity is fixed to 3 (6) units of the asset, a feature which is built into GIMS by default and only requires the setting of a parameter. In treatments CR100, CR150 and FLX, this capacity depends on a subject’s cash balance relative to the dividend holding value of the assets sold short. This would require an additional “checker” in GIMS, the implementation and testing of which we estimate to require 1–2 h work. Another interesting design feature is that Haruvy and Noussair (2006) roll a die to determine the amount of dividend to be paid. While not pre-configured, the built-in experimenter screens render it very easy to implement such a functionality in GIMS, such that we would estimate a time requirement of 30–60 min.

#### A.2.5 Cheung and Palan (2012)

Cheung and Palan (2012) is an interesting paper in terms of the features required of the asset market software, because it reports results from both continuous double auction markets and sealed bid–ask call auction markets. Still, either treatment can readily be run using GIMS, without any need to modify the programming beyond adapting the exit questionnaire. In fact, GIMS’ call auction price determination and trade settlement mechanisms are considerably more efficient than those employed in Cheung and Palan (2012).

#### A.2.6 Kirchler et al. (2012)

Kirchler et al. (2012) (like Noussair et al., 2001) implement different fundamental value trajectories using combinations of positive and negative dividends payable at the end of every period, plus a terminal dividend payable after the last period of trading. GIMS offers this functionality by default. In some treatments the dividend income is added to subjects’ cash balance immediately, while in others it accrues to a savings account which cannot be tapped to fund trading, but counts towards subjects’ final payoff. While the first is the standard in GIMS, the latter would require approximately 30 min of programming and testing to implement. Furthermore, in some treatments Kirchler et al. (2012) grant periodic, deterministic cash inflows to their subjects, while in others they deduct periodic, deterministic cash outflows from their cash balances. Mimicking these in GIMS would require minimal additional programming of about 15 min.

The market architecture in Kirchler et al. (2012) consists of a continuous double auction with open order books where all orders are executed honoring price–time priority. They permit multiple unit trading and the partial execution of limit orders, while disallowing short selling and margin buying. Their trading screen shows a real-time chart in addition to displaying subjects’ cash and asset balances. All of these features are GIMS defaults. They also elicit the Holt and Laury (2002) risk measure from their subjects after the conclusion of the 10 periods of trading in the market. As noted in the body of the present paper, GIMS is endowed with the built-in capability of eliciting this measure of subjects’ risk preferences.

#### A.2.7 Sutter et al. (2012)

In Sutter et al. (2012), the market features a continuous double auction with an open order book. Subjects can buy and sell, but neither sell short nor buy on margin. Wealth is carried over between each of the ten periods constituting one round, but not between each of the three rounds in a session. Subsets of all subjects receive information about the dividend to be paid in the current or in the current and the upcoming period. Furthermore, the dividend realizations were drawn once and then reused in multiple markets. This functionality is offered in GIMS by default, except for providing the information to subjects, which can be expected to require 30 min to program. Furthermore, Sutter et al. (2012) display previous periods’ mean prices on a results screen following the end of the period. This can also be implemented in a few minutes’ work.

#### A.2.8 Kleinlercher et al. (2014)

Kleinlercher et al. use a continuous double auction market with an open order book. Their main design feature with regard to their research question is the implementation of different incentive schemes, which is trivial to do in GIMS (it requires changing a single line of code). The most demanding element of their design in terms of the experimental software is that the authors allow subjects to trade in two markets simultaneously (aligned side-by-side on the screen). GIMS does not currently offer this option. While some preparations for multiple market trading are already implemented (e.g., all offers and orders carry a market ID identifying the market they belong to), implementing this feature set would require duplicating the trading screens and changing the programming of some market-wide variables (e.g., the last price). Including testing, we would expect this to require 1–3 days’ work.

## Footnotes

1 Data as of 2012; see World Federation of Exchanges (2013).

2 Take for example z-Tree (Fischbacher, 2007), probably the most widely used experimental software platform in economics. z-Tree automatically records the times when subjects click buttons in an experiment. However, it does so with a precision of only 1 s, and it automatically records only the last time a button was pressed on a given screen of an experiment. Furthermore, it does not record events like subjects’ movement from one stage of the experiment to another. While it offers the possibility of programming routines to record these events, hardly any of the programs underlying published papers make use of it, thereby limiting researchers’ scope of analysis.

3 Note that we suppress the initial “http://” for all internet links in this paper in the interest of conserving space.

4 Information from EconPort (2006) and from an email exchange with the developers.

5 Information from Flex-E-Markets (2014).

6 Information from Caltech (2014).

7 Information from Rotman School of Management (2014) and from an email exchange with the developers.

8 Information from SoPHIE Labs (2014) and from an online presentation by the developers.

9 Strictly speaking, z-Tree continuously records interim data in a so-called gamesafe file. Since this file is not designed to be human-readable, however, retrieving data from it in the event of a crash is difficult.

10 See Palan (2014).

11 This includes the possibility to allow trading in derivative securities. A dark pool or even over-the-counter trading would have to be implemented in a similar way, but would only allow for participation of a subset of all traders.

12 When a limit is set on the maximum number of concurrent outstanding bids and asks, subjects have to manually cancel one or more of their orders in order to submit new ones in case they hit the limit.

13 Neither dividends nor signals have to be implemented.

14 Assumptions: three limit bids and asks, respectively, and one market bid and ask, respectively, per trader. Each price between 1 and the number of limit bids/asks (72) is used once; each order’s volume is randomly drawn from the integers in the interval [1,100]; submission times randomly drawn from the interval [0,1]. Run on an Intel^®^  Mobile Core™ i7-2630QM.

15 As a side note, using a standardized software package also reduces the number of design details which need to be reported in papers, thus shortening the experimental design section and focusing the reader’s attention on the relevant aspects of the experiment, namely the treatment variation.
